# Supplementary material for: Are carbon emissions trading and green financial instruments synergistic? -Comprehensive quantitative research based on content analysis
Source: PLoS One. 2024 Mar 7;19(3):e0298601. doi: 10.1371/journal.pone.0298601 (PMC10919601; doi:10.1371/journal.pone.0298601)
Supplement: S1 Data — (ZIP) [file pone.0298601.s001.zip › Data/地级市Z_F工作报告.zip!/╦─┤¿/│╔╢╝╩╨/└┤╫╘╣1⁄2╓┌║┼í╛┬φ┐╦╩2╛▌═°í┐_2017.docx]

各位代表：

我代表市人民政府向大会报告工作，请予审议，并请市政协委员和其他列席人员提出意见。

一、2016年工作回顾

2016年是实施“十三五”规划开局之年。在党中央国务院、省委省政府和市委的坚强领导下，在市人大、市政协的监督支持下，我们紧紧依靠全市人民，全面履行政府职责，主动作为、开拓创新，全市经济社会继续保持良好发展态势。预计全年实现地区生产总值1.2万亿元，增长7.5%；固定资产投资8370亿元，增长14.3%；一般公共预算收入1175亿元，增长7%；社会消费品零售总额5620亿元，增长10%；城乡居民人均可支配收入分别增长8.1%、9.9%，居民消费价格上涨2%。重点抓了九个方面工作：

（一）大力实施创新引领行动。统筹推进全面创新改革试验，全社会研发投入增长12.7%，发明专利申请量突破3.6万件、增长20.8%，新登记市场主体30.4万户，新增科技型企业1.2万家，分别增长32.7%、9.1%。深化政产学研用协同创新，制定科技成果转移转化“新十条”政策，在全国率先推出“早确权、早分割、共享制”改革模式，与7所在蓉高校院所签署共建“双一流”战略合作协议，设立5个新型产业技术研究院。系统推进军民融合创新发展，建设成都信息安全产业园等102个项目、总投资1431.1亿元，加快建设航空整机等6个省级产业基地、天府新区军民融合创新产业园等6个市级产业园。国家自主创新示范区全面落实“6+4”先行先试政策，国家技术转移西南中心成功落户。成都科学城引进中科院成都科学研究中心等10个校院地合作项目，清华能源互联网研究院等15家科研企业入驻。深入实施“创业天府”行动计划，成功举办2016中国成都全球创交会，举办“菁蓉汇”走进韩国、以色列等系列活动180场，郫都区、中国电信（成都）、四川大学获批全国首批双创示范基地，新增国家级众创空间31家、孵化器面积102万平方米。大力优化创新创业环境，出台人才新政十条，引进3个诺贝尔奖团队，新建20个院士专家工作站，获批国家知识产权质押融资和专利保险双示范城市。

（二）大力实施工业强基行动。坚持把工业作为经济发展的重中之重，加快推进162个重大技改项目，新增国家企业技术中心4户，预计完成工业投资2185亿元、技改投资1850亿元，分别增长37.1%、38.5%；工业增速持续回升，预计规模以上工业增加值增长7.4%。深入开展工业“竞进拉练”，东风神龙、英特尔骏马等135个项目竣工投产，京东方第6代AMOLED生产线、中铁轨道交通等项目加快建设，中电子8.6代液晶面板、中电科信息安全等119个项目开工建设，紫光IC国际城、吉利新能源汽车等139个重大项目签约引进。产业转型升级加快，电子信息、汽车等优势产业做大做强，电子信息产业规模达4800亿元，汽车整车产量突破110万辆、产值达1700亿元；轨道交通、航空航天、生物医药、新材料等战略性新兴产业加快培育，产值分别增长10%、40%、12%、18%。工业园区布局优化，确立“3+N”工业发展格局，规划“大创造”“大智造”“大车都”工业板块，推进高新区、经开区拓区和区（市）县工业园区建设。强化企业服务保障，新增规模以上企业334家，产值过百亿工业企业达10家；推广中小微企业“壮大贷”、民营企业应急周转金等模式，中小微企业贷款余额增长11.1%。

（三）大力实施服务业提升行动。加快国家服务业核心城市建设，预计服务业增加值增长8.5%，完成内贸流通体制改革试点，获批建设自贸试验区，被列入国家服务贸易创新发展试点。积极打造西部金融中心，启动建设成都天府国际基金小镇，设立规模400亿元的成都前海产业投资引导基金，新增A股上市公司7家、新三板挂牌企业106家，本外币存款余额3.2万亿元、贷款余额2.5万亿元，成都金融资产交易中心正式运营，金融综合竞争力位居中西部第一。加快建设世界旅游目的地城市，引进华侨城等重大文旅项目112个，先后在30多个境外城市开展旅游营销，入选全球“2017年21个必去旅游目的地城市”，接待游客2亿人次，旅游总收入达2500亿元、增长22.6%。加快建设国际美食之都，成功举办旧金山成都美食文化节，荣获2016中餐国际化推广示范城市奖。努力建设国际购物天堂，着力提升春熙路盐市口等商圈业态，新增离境入境退税商店41个，全球80%以上的知名品牌落户成都，电子商务交易额达8000亿元，获批国家跨境电商试点城市。加快建设国际会展名城，成功举办G20财长和央行行长会议、世界航线发展大会、西部金融中心论坛、第四届中国网络视听大会、第七届中国博物馆博览会等重大展会530场次。现代物流、科技服务、检验检测、信用评级等生产性服务业加快发展。

（四）大力实施融入“一带一路”行动。深入实施“蓉欧+”战略，启动国际铁路港建设三年行动计划，蓉欧快铁中线延伸至欧洲更多城市，新开通北线至莫斯科、南线至伊斯坦布尔班列，初步形成“三线并进”格局，全年开行500列，居中欧班列首位；稳定开行至上海、深圳等8个国内城市互联互通班列，带动联想、戴尔等企业加速向成都转移适欧产能。天府国际机场征地拆迁基本完成并开工建设，启动双流国际机场扩能改造，新开通至悉尼、马德里等6条国际定期直飞航线，国际（地区）航线达95条，客货吞吐量分别突破4500万人次、58万吨。“四主三辅”铁路客运枢纽场站加快形成，川藏、成贵、西成、成兰等铁路加快建设，成德铁路实现公交化开行。成都经济区环线高速公路加快建设，成渝西昆贵“钻石经济圈”高速公路主骨架加快构建。公路口岸（新址）、铁路保税物流中心（B型）封关运营，陆运进口肉类、空运进口水果等指定口岸建成投用，获批国家平行进口汽车试点城市。全面实施“153”重大项目引进计划，签约引进重大项目270个，其中30亿元以上先进制造业项目17个，在蓉世界500强企业达278家，实际利用内资4100亿元、外资85.3亿美元，分别增长14.5%、13.4%。中德、中法、中韩、中古、新川等国际合作园区加快建设。编制企业“走出去”五年行动计划和“一带一路”产业指导目录，新增备案境外投资企业149家，进出口总额增长5%以上。新增瑞士驻蓉总领事馆，驻蓉领事机构增至16家，国际友城增至32个，荣获中国国际友城交流合作奖。大力推进“1+7”成都平原城市群同城化发展，与雅安、资阳、甘孜、凉山达成合作协议，促进成渝经济区一体化发展，推动成渝西昆贵“钻石经济圈”协同发展，与青海签署省市合作备忘录，加强与长三角、珠三角、京津冀等区域城市合作。

（五）大力实施城建攻坚行动。获批建设国家中心城市，编制城市总体规划（2016-2030年）和国家中心城市建设行动纲要（2016-2025年），启动2049远景发展战略规划研究。加快中心城区有机更新，启动成都中心建设和三环路扩能提升，加快推进小街区规制示范区建设，完成城中村改造4650户、棚户区改造1.28万户，实现“北改”投资720亿元。统筹推进天府新区基础设施、产业园区和城市组团建设，西部国际展览中心主体竣工，益州大道南段等31个项目开工建设，“六纵六横”骨干路网体系基本形成。加快4个小城市和10个特色小城镇建设，德源镇、安仁镇获评第一批中国特色小镇，“双核共兴、一城多市”网络城市群和大都市区格局加快构建。大力推进轨道交通加速成网，地铁3号线一期、4号线一期开通运营，5号线等7个项目加快建设，6号线等6个项目开工建设，地铁运营和在建里程分别达108公里、276公里，第三期轨道交通建设规划获批，新一轮线网规划编制完成。优化中心城区路网体系，成温邛快速路和大件路外绕线建成通车，天府国际机场高速公路开工建设，成彭高速公路扩能改造加快推进。新建4G基站7300多个。预计完成城建投资1880亿元。

（六）大力实施美丽成都行动。启动“宜居水岸、活水成都”工程，开工建设李家岩水库，加快环城生态区建设，青龙湖一期、桂溪生态公园等建成开放，积极开展海绵城市建设，获批全国地下综合管廊试点城市。启动大规模绿化全川成都行动，实施“增花添彩”工程，规划建设60个赏花基地，新增公园绿地13万平方米，中心城区绿化覆盖率达40.9%，全市森林覆盖率达38.7%。积极防治大气污染，实施减排、压煤、抑尘、治车、控秸五大工程，大气主要污染指标持续下降，空气重污染天数比上年减少9天。提升水环境质量，实施“河长制”管理，持续开展岷江、沱江流域水环境治理，推进城市建成区黑臭水体综合治理、下河排水口污染治理、污水处理厂扩能提标改造，岷江流域水质明显改善。提升城市治理水平，制定80项城市管理标准，开展城郊结合部环境治理攻坚行动，强化生活垃圾分类处理，实施市政设施提升工程，城乡人居环境持续改善。

（七）切实加强重点领域改革。制定供给侧结构性改革“1+5”实施方案，淘汰落后产能企业51户，房地产市场保持平稳健康发展，规模以上工业企业资产负债率下降2.4个百分点，降低电价和天然气价格，阶段性降低社保费率，为企业减负近70亿元。深化“放管服”改革，取消调整行政审批56项，行政审批前置条件及中介服务事项同比分别减少33%、76.6%。深化商事制度改革，实施企业“五证合一、一照一码”和个体工商户“两证整合”登记改革。深化财税和投融资体制改革，推出PPP项目39个，首批“拨改租”项目签约总投资255亿元，7支企业债获准发行，争取国家专项建设基金367.4亿元，“营改增”有序推进，民间投资增长15.5%、占投资比重达56.2%。完善国有企业监督管理体制，推进3户国有资本投资运营公司试点，5户市属国企在新三板或区域股权交易市场挂牌。深化统筹城乡改革，基本完成农村“新四权”登记颁证，启动不动产统一登记，试点集体资产股份化改革，探索农户自愿有偿退出宅基地途径，新建成“小组微生”新农村综合体100个。顺利完成简阳市代管关系变更和工作并轨对接，双流县、郫县撤县设区。农业发展方式加快转变，都市现代农业稳中提质，“米袋子”“菜篮子”工程深入实施，农产品生产区、加工区、乡村旅游区融合发展，地理标志产品达61个，预计农业增加值增长3.9%。

（八）切实加强社会事业发展。坚持民生优先，全面完成十大民生实事，全市财政民生支出1045亿元，占一般公共预算支出67.5%。突出抓好简阳市脱贫攻坚，完成2965人易地扶贫搬迁，31个省定贫困村、15952人实现摘帽脱贫。高标准推进城乡扶贫开发，实施“百村万户”精准扶贫，相对贫困村农民人均可支配收入达1.2万元，增长20%以上。加强甘孜、阿坝19个县对口支援，实施产业发展、公共建设、教育医疗等重点项目116个。全面完成“4·20”芦山强烈地震灾后恢复重建和对口支援雅安任务。实施积极的就业政策，城镇新增就业27.9万人，城镇登记失业率预计在4%以内。实施全民参保计划，大病医疗互助补充险最高支付比例提高至90%，发放困难群众价格补贴2.3亿元。竣工保障性住房11572套。医药卫生体制改革加速推进，新型医联体实现基层全覆盖，分级诊疗模式初步形成，中医药事业健康发展，全面两孩政策平稳实施，获批全国首批健康城市试点。大力发展教育事业，建成公办幼儿园206所，新增学位5.9万个，投入21亿元实施义务教育免费、中职教育学费减免等助学工程，妥善解决36.5万名进城务工人员随迁子女接受义务教育，高考本科升学率突破70%，市属高校教育质量稳步提升。大力发展文化体育事业，开展文化惠民演出3000余场次，开工建设城市音乐厅，建成并开放成都博物馆，被列入中国“十大古都”，初步完成奥体中心规划选址，成功举办ATP250世界巡回赛、第十三届市运会等体育赛事。连续七次获评“全国双拥模范城”。出台养老服务促进条例，建成社区养老院72个，改筹建养老床位1.75万张，为7.38万名困难家庭失能老人和80周岁以上老人提供居家养老服务。食品药品安全形势持续稳中向好，在全国率先探索出全过程控制、全链条信息化的食品药品智慧监管模式。扎实推进平安成都建设，完善立体化社会治安综合防控体系，实施重点行业领域安全隐患专项整治，安全生产事故持续下降。启动“七五”普法，大力推进信访法治化建设，社会保持和谐稳定。连续5年获评“中国最具幸福感城市”。

（九）切实加强政府自身建设。深入推进依法行政，严格执行市人大及其常委会决议决定，主动接受法律监督和工作监督，自觉接受市政协民主监督，全力做好人大代表和政协委员建议、提案办理，累计办结建议、提案1309件，办复率100%。成立市政府国际咨询顾问团，建立市民参事制度。深化政务公开，公开政务信息150万条，集中公开188个便民服务电话。深入开展“两学一做”学习教育，严格落实从严治党主体责任、党风廉洁建设各项规定，持续严管“三公”经费，完成机关公务用车改革。

国家安全、国防动员、防灾减灾、应急管理、消防、人防工作切实加强，民族宗教、港澳台、妇女儿童、老龄、残联、慈善工作取得新进步，审计、统计、档案、保密、气象、邮政、参事、地方志、哲学社会科学工作取得新进展。

各位代表，过去一年，全市干部群众开拓奋进，社会各界对政府工作大力支持。在此，我代表市人民政府，向全市人民，向人大代表、政协委员、各民主党派、工商联、无党派人士、各人民团体，向中央和省驻蓉单位，向所有关心支持成都改革发展的同志们及各界人士，表示衷心的感谢！向人民解放军指战员、武警官兵、政法干警和民兵预备役人员，表示崇高的敬意！向港澳台同胞、广大侨胞和海内外朋友们，表示诚挚的谢意！

我们也清醒地认识到：发展不足和资源环境制约依然是成都最大的实际，保持经济较快发展面临较大压力；产业核心竞争力不强，创新驱动尚未成为发展主引擎，新旧动能转换、转型发展的任务还很重；在更宽领域、更高层次参与全球资源要素整合的能力还不强，国际竞争力还有待进一步提升；大气、水、土壤污染防治形势严峻，系统推进绿色低碳发展的措施还不够；交通拥堵等“大城市病”比较突出，就业、教育、医疗等公共服务供给与人民群众的期盼还有较大差距；政府职能转变仍需深化，依法行政能力、政务服务水平有待提升，营商环境还需优化；少数公职人员廉而不勤、勤而低效，思想解放不够、专业水平不高，运用互联网等现代化手段服务群众、治理城市的能力不足。对这些矛盾和问题，我们一定千方百计加以解决，不辜负全市人民的期盼！

二、2017年工作建议

2017年，是我国政治生活中具有重要意义的一年，也是我市推进国家中心城市建设、实施“十三五”规划和深化供给侧结构性改革的关键一年。做好政府工作的基本思路是：全面贯彻党的十八大和十八届三中、四中、五中、六中全会精神，深入学习贯彻习近平总书记系列重要讲话精神，按照中央“五位一体”总体布局、“四个全面”战略布局，落实省委“三大发展战略”和省政府部署，围绕市委“157”总体思路，坚持以新发展理念为指引，以适应把握引领经济发展新常态为大逻辑，以深化供给侧结构性改革为主线，以“稳中求进”为工作总基调，以“六大行动”为抓手，始终保持专注发展、转型发展战略定力，深入实施系列2025规划，全面做好稳增长、促改革、调结构、惠民生、防风险各项工作，努力保持经济平稳健康较快发展和社会和谐稳定。

2017年经济社会发展的主要预期目标是：地区生产总值增长7.5%左右，全社会固定资产投资9200亿元，一般公共预算收入增长7.5%，社会消费品零售总额增长10%，城乡居民收入与经济增长同步，城镇登记失业率控制在4%以内，居民消费价格指数调控目标控制在103以内，万元地区生产总值能耗进一步降低。重点抓好以下九个方面工作：

（一）着力构建现代产业体系，加快建设西部经济中心

一是加快发展先进制造业。坚持工业“一业定乾坤”，持续做大做强工业经济，围绕建设国家重要先进制造业中心，争创“中国制造2025”试点示范城市，深入实施工业强基行动，完成工业投资2850亿元、增长30%以上，占固定资产投资比重30%以上，力争新增规模以上企业220户，规模以上工业增加值增长7.5%。规划建设龙泉山东侧工业发展走廊，加快建设“大创造”“大智造”“大车都”工业板块，加强都市组团和卫星城工业园区建设，加快形成“3+N”工业发展格局。持续开展工业“竞进拉练”，开工建设吉利新能源汽车、四川航天高新技术产业园等123个项目，加快建设中电子8.6代液晶面板、科伦生物医药、成都芯谷等151个项目，推动京东方第6代AMOLED生产线、中电锦江等158个项目竣工投产。提升产业发展质量，优化发展电子信息、汽车等优势产业，培育壮大轨道交通、航空航天、生物医药、智能制造装备等战略性新兴产业，推动产业集群成链发展，加快培育万亿级产业集群；改造提升轻工、食品、建材等传统产业，推动老工业基地转型升级，支持工业设计、创意咨询等专业机构和行业商（协）会发展，大力推广智能制造、绿色制造和服务型制造，开展数字化车间和智能工厂应用试点示范，建设更多“智慧工厂”。

二是提升发展现代服务业。着力提高生产性服务业比重，优化生活性服务业结构，培育新兴消费模式，提升服务业标准化发展水平，服务业增加值增长8%。加快西部金融中心建设，深入实施经济证券化行动计划，大力发展科技金融、绿色金融、普惠金融和消费金融，力争新增上市公司8家、新三板挂牌企业70家，区域股权交易市场挂牌企业总量达1200家。加快打造世界旅游目的地城市，推动航空大世界、万达文化旅游城等重大旅游项目建设，打造龙门山（大青城）和“三湖一山”生态旅游功能区，推动旅游景区提档升级，用好大熊猫、美食两张名片，大力开展城市全球营销，力争游客总数达2.1亿人次。加快打造国际购物天堂，实施“商圈提升年”行动，推出创意经典“成都礼物”，深入推进国家跨境电子商务综合试验区建设。加快打造国际会展名城、国际赛事名城，办好联合国世界旅游组织大会、国际乒联世界巡回赛等展会赛事。

三是优化发展都市现代农业。深化农业供给侧结构性改革，增加绿色优质农产品供给，狠抓农产品标准化生产、品牌创建和质量安全监管。推动农业集约式发展，加快实施100万亩高标准农田工程，推进“都市农业创新创业园区+特色小镇”建设，抓好“现代农业产业园区+小组微生新农村综合体”建设，大力推广“土地预流转+履约保证保险”、农业共营制等模式。推动农业经济型提升，继续抓好“米袋子”“菜篮子”工程，完善农产品物流体系，发展农产品精深加工，提升“天府源”公用品牌价值，打造国家都市现代农业示范城市、世界乡村旅游目的地。加快发展智慧农业、低碳农业，积极推进农村电子商务。农业增加值增长3.3%。

四是大力发展新经济。加快发展大数据经济、智慧经济、分享经济、创意经济等新业态，抢占经济新高地。加快发展众创、众包、众扶、众筹等新模式，形成增长新动力。加快发展物联网、人工智能、虚拟现实、增强现实等新技术，重塑产业链、供应链、价值链，拓展发展新领域。力争新经济指数进入全国前五名。

（二）着力实施创新驱动发展战略，加快建设西部科技中心

一是深入推进政产学研用协同创新和军民融合创新。推进政府科技管理方式改革，推广职务科技成果混合所有制改革，新建4个新型产业技术研究院，全社会研发投入290亿元，新增科技型企业1.2万家以上，实现高新技术产业产值8700亿元。创新军民融合体制机制，搭建转化应用推广平台，设立规模达20亿元的军民融合产业投资基金和创新创业基金，加快建设30个投资10亿元以上的重大项目，不断完善“1+N”产业发展体系和空间布局体系。

二是深入推进国家自主创新示范区建设。推动出台示范区条例，发布实施示范区发展规划纲要，着力打造欧洲中心等四大国家级平台，加快建设天府国际生物产业城，按照“一区多园”模式，争取更多先行先试政策，打造制度创新和模式创新试验田。

三是深入推进成都科学城建设。加快推进国家重大科技基础设施等创新平台（基地）建设，引进培育新型孵化转化载体10家以上，加快建设中科院成都科学研究中心等重大科学项目，积极推动北航西部国际创新港等10个技术创新项目入驻，引进国家工程实验室2家以上，争创国家科学中心和国家技术创新中心。

四是深入推进“创业天府”行动计划。深化国家双创示范基地建设，打造“创业天府”云孵化平台，办好2017中国成都全球创交会，开展“菁蓉汇”走进德国、俄罗斯等系列活动180场，吸引更多全球创新创业要素加快向成都集聚。大力弘扬创新文化，搭建大众创意共享转化平台，营造人人愿创新、创意能实现的社会氛围。

五是深入推进四大平台建设。做强政策支撑平台，修订科学技术进步条例，加大优秀科技人才引进培养力度，支持高校院所科技人才兼职或离岗转化研发成果。做强双创孵化平台，优化提升以菁蓉国际广场、天府菁蓉中心和菁蓉镇为引领的“3+M+N”创新创业空间布局，新增孵化器30家、面积50万平方米。做强开放合作平台，加快国家级海外人才离岸创新创业基地、中韩创新创业产业园等平台建设。做强成果转化平台，提升发展环高校成果转化区，加快建设国家技术标准创新基地，争创国家知识产权强市。

（三）着力加强城市文化建设，加快建设西部文创中心

一是大力繁荣文化事业。规划建设成都大剧院、自然博物馆、成都美术馆、群众文化活动中心、三国蜀汉城等重点文化设施，推动国家公共文化服务体系示范区提档升级。推动文艺创作生产繁荣发展，开展文化艺术进家庭、进社区等文化惠民活动。倡导全民阅读，建设书香成都，着力营造蜀风雅韵的文化氛围。

二是大力发展文创产业。加快发展音乐、影视、动漫、游戏等产业，着力培育数字娱乐、数字传媒等新业态，培养引进文创优秀人才，优化文创产业结构，提升文创贸易服务，繁荣文化市场，文创产业增加值达650亿元。加快建设“音乐之都”，打造国际性国家级音乐生产地、音乐设施设备集散地、版权交易地和演出聚集地，提升“成都国际音乐诗歌季”等品牌影响力，音乐产业产值增长12%。搭建文创产业平台，建设文创产业国家重点实验室，打造中国西部文创产业园、中国西部音乐谷、少城文创硅谷等文化创意产业园。

三是切实强化文化遗产保护。加强历史文化遗址和建筑保护，打造东华门、邛窑等5个大遗址公园，建设金沙遗址等城市文化核心区，优化提升琴台路业态，提升宽窄巷子、锦里等文化街区品质，推动古蜀文明遗址、“蜀道”申报世界文化遗产。依法加强非物质文化遗产传承保护，积极推进非遗生活化、产业化，弘扬川剧、清音等地方特色文化，加强蜀锦、蜀绣等传统工艺保护利用，办好第六届中国成都国际非遗节。

四是持续扩大文化交流。实施文化“走出去、引进来”升级计划，在摩洛哥建设文化中心。加强城市文化营销推广，打造大熊猫文化、音乐诗歌文化、南丝路文化等城市文化名片，巩固提升“博物馆之都”“非遗之都”等国际文化品牌，办好成都文化周等国际文化交流活动，推动成都文化融入世界、世界文化走进成都。

（四）着力提升双向开放水平，加快建设西部对外交往中心

一是全面建设自贸试验区。坚持以制度创新为核心，制定三年行动计划，打造具有“一带一路”特色的内陆自贸区。推动投资便利化，推行外商投资“准入前国民待遇加负面清单”管理模式，完善外资监管服务体系。推动营商环境法治化，建立与国际贸易规则接轨的行业监管和资格审查制度。推动服务贸易自由化和货物贸易便利化，加快金融服务创新，建设国际贸易“单一窗口”，全面推进“一站式作业”。

二是不断提高经济合作水平。围绕“153”重大项目引进计划，着力招大引强、招高引新，强化“一把手”招商引资和“出勤率+签约率+开工率”综合考核，新签约引进重大项目300个以上，其中投资100亿元以上项目6个、50亿元以上项目7个、30亿元以上项目15个，实际到位内资4400亿元、外资93亿美元。鼓励企业“走出去”，加强与“一带一路”沿线国家产品、技术、服务等标准互联互通互认，拓展“成都制造”“成都服务”“成都品牌”国际市场空间。提升中德、中法、中韩、中古和新川等国际合作园区承载能力。促进对外贸易“优进优出”，进出口总额增长7%。

三是加快拓展对外交流合作。积极开展国际友好交流，推动更多领事机构入驻成都，加强与国际机构交流合作，发展更多国际友好城市。办好东亚地方政府会议、城市可持续发展高层论坛等国际交往活动。加快建设成都领馆区，深入实施外籍人士“家在成都”工程，强化涉外医疗、教育、法律等公共服务保障。

四是大力推动区域协同发展。强化首位城市责任，深化“1+7”成都平原城市群同城化发展，深入推进成德绵协同创新共同体，全面落实成攀、成阿、成甘、成凉等区域合作协议。推动成渝城市群一体化发展，促进建立成渝西昆贵“钻石经济圈”协商合作机制，加强与长三角、珠三角、京津冀等地区城市务实合作。

（五）着力构建立体交通体系，加快建设西部综合交通枢纽

一是深入推进“蓉欧+”战略。加快构建“两港三网”，促进贸易发展、服务业提升、产业集聚和国际产能合作。持续优化蓉欧快铁中线班列，稳定开行南线和北线班列，力争全年开行1000列以上。加快建设成都国际铁路港，增设天府新区（新津）铁路口岸，打造“蓉欧+”陆港产业园和临港经济示范区，吸引更多对欧洲和中亚、适宜铁路运输的高附加值产能向成都转移聚集。加快天府国际机场和机场高速等场外交通体系建设，启动建设天府国际机场临空经济区，加快双流国际机场扩能改造，力争实现7×24小时通关运营，织密洲际航线网和国内干线网，国际（地区）航线达98条，力争客货吞吐量分别突破4800万人次、62万吨。

二是打造国家高铁和区域高速公路枢纽城市。建成成昆铁路扩能工程成都至峨眉段，加快成蒲、西成、成兰、川藏和成贵铁路建设，加快推进蓉昆高铁成自段、成都至西宁、至格尔木铁路前期工作。建成成安渝高速成都段，加快成都经济区环线高速建设和成彭高速扩能改造，开工成宜、成资潼高速建设和成乐高速扩能改造。加快国家多式联运示范工程建设。

三是加快完善市域交通体系。围绕“650+”目标，积极推进轨道交通加速成网，加快建设地铁5号线、6号线、18号线等10个项目，开通运营地铁7号线、10号线一期、4号线二期，实现运营里程179公里、在建里程329公里，加快都江堰旅游客运专线等有轨电车项目建设。鼓励发展空铁、磁悬浮列车等新制式轨道交通。加快大都市区和市域铁路公交化改造，推动高铁、市域铁路、地铁三铁融合。实施城市路网提升工程，加快构建中心城区和天府新区双核高快速路网，启动成环路等市域快速路建设，加快三环路扩能提升，推动成渝高速入城段、日月大道、成洛路等道路改造。

（六）着力优化城市功能品质，加快建设宜业宜居城市

一是加强城乡科学规划。争取城市总体规划获批，完成2049远景发展战略规划研究和“十三五”重点专项规划编制，修编“十三五”规划纲要和系列2025规划，出台国家中心城市专项实施方案和年度计划。完善规划实施机制，建立健全空间基础信息平台和发展战略数据库，着力构建土地利用全生命周期管理体系。深入开展城市资源环境承载力研究，划定城市开发边界和生态红线，科学制定城市中长期人口规划，优化人口结构和质量，控制中心城区人口过快增长，避免城市人口无序增长，实现人口、资源、环境协调发展。统筹推进市域城镇体系协调发展，全面提升都市组团和卫星城综合承载能力，重点打造4个小城市和12个特色镇，推动简阳市全面一体化融入成都，加快构建“双核共兴、一城多市”网络城市群和大都市区发展格局。

二是推进中心城区有机更新和天府新区建设。疏解中心城区传统产业功能，加快推动府河桥、荷花池等老旧市场调迁改造升级。深入实施“北改”工程，加快火车北站等6个重点片区改造，年度总投资400亿元。完成改造中心城区棚户区5200户、老旧院落550个。深入实施小街区规制，全面推进9个示范区建设和18个街区整治。加快打通“断头路”，完善中心城区“3900+1200”路网体系，推动中心城区与近郊区道路互联互通。加快天府新区建设，着力推进成都科学城、天府商务区、西区产业园等重点区域城市建设，加快地铁中心站等节点枢纽和天府环线等交通干线建设，开工建设国际会议中心、奥体中心等10个重大项目。

三是增强基础配套功能。加快推进成都中心、成都科学馆、城市音乐厅、音乐坊建设，建成投用成都演艺中心、城市南部片区青少年宫，开工建设成都国际足球中心。启动中心城区居住区、社区公共配套设施建设三年攻坚行动，建设公共配套设施项目400个，着力形成“15分钟公共服务圈”。建成自来水二期净水厂工程，鼓励试点管道直饮水供水。深入推进海绵城市建设，完成21个地下综合管廊试点项目，探索深层地下空间综合开发。加快建设变电站、公厕、垃圾转运站、环卫工人作息房等设施，强化基础配套设施使用管理。加快“宽带中国”示范城市建设，推进互联网骨干直联点和城域网出口带宽扩容，争取率先开展5G试点，实施宽带网速200M普及行动，平均上网带宽超过50M，让成都连接世界的速度更快。

四是强化城市综合治理。推动城市综合管理条例等法规出台，健全环境卫生、市容秩序、道路桥梁、景观照明等标准和规范。加强城市智能感知系统建设，推进大数据、物联网等技术运用，拓展数字城管功能，提升城市管理问题的发现率和处置率。提高小区物业管理覆盖率和服务水平，深化城郊区域环境治理攻坚行动。

（七）着力推进绿色低碳发展，加快建设美丽中国典范城市

一是坚决打好大气、水、土壤污染防治“三大战役”。重点抓好扬尘、机动车尾气、工业废气和臭氧污染治理，全部淘汰黄标车，持续做好禁煤禁烧禁放工作，强化重点污染源实时在线监测，确保自动监测设备运行率100%、传输有效率达95%以上，向社会公布污染源重点监控企业名单，强化第三方机构和市民监督，建立区（市）县大气质量月排名和改善奖惩机制，主要污染物PM2.5浓度降低4.8%，重污染天数持续减少。全面实行“河长制”管理，强化毗河、江安河等重污染流域治理，推动东风渠综合整治，加强岷江、沱江流域跨界断面水质监测考核，完成城市建成区黑臭水体综合治理，深化农村面源污染治理。制定土壤污染防治行动计划，完善土壤监测网络，强化农业生产化肥和农药减量控害，开展土地重金属污染治理和修复，保护提升耕地质量。我们将严肃认真地打好“三大战役”，努力让市民呼吸到更清新的空气、喝上更干净的水、吃上更放心的食物。

二是完善绿色低碳发展制度体系。强化企业环保主体责任，深化环境污染责任保险试点，推进企业环境信用评价体系建设，加大环境违法行为执法力度，依法严厉打击偷排漏排、篡改伪造监测数据、非法处置危险废弃物等各类环境违法行为。建立生态补偿制度，推动环保机构监测监察执法垂直管理制度改革。强化绿色低碳发展政绩考核问责，实行领导干部自然资源资产离任审计，建立生态环境损害责任终身追究制。全面落实耕地和基本农田保护主体责任，健全永久基本农田“划、建、管、护”长效机制，强化农业、林业生产能力和生态功能。

三是加快构建绿色低碳能源体系和城市体系。提升电力、天然气清洁能源消费比重，切实减少煤炭消费量；开展全域限煤行动，推动工业企业煤改电、煤改气，推广运输工具油改电、油改气，鼓励发展天然气分布式能源；大力推动电动汽车消费，率先推动公交车、公务用车电动化，加快建设充电站（桩）设施，逐步减少化石能源使用。坚持把绿色低碳理念融入城市规划建设管理全过程，突出职住平衡，切实减少潮汐交通现象；积极推广绿色建筑、装配式建筑和成品建筑，减少使用玻璃幕墙，全面推广绿色施工，实施建筑垃圾现场资源化利用；建立行人、非机动车和公交车优先的路权体系，完善城市慢行系统，试点建设自行车高速公路，鼓励支持共享单车、共享新能源汽车等共享交通健康发展。

四是建设城市碳汇能力体系。加快中心城区公园和绿地建设，持续抓好“增花添彩”“宜居水岸、活水成都”等重点工程。全面推进环城生态区建设，加快推进江安湖、青龙湖二期、北湖等水库建设，加快建设李家岩水库，推进三坝水库、久隆水库前期工作。深入实施大规模绿化全川成都行动，规划建设龙泉山城市森林公园和大熊猫国际旅游目的地，加快构建“两山两环、两网六片”的生态安全格局，全市森林覆盖率达39%。

五是完善绿色低碳消费体系。抓好低碳城市试点建设，倡导绿色低碳文化，鼓励绿色低碳消费，推广绿色办公，倡导绿色低碳出行，营造绿色低碳生活消费的社会氛围。启动节能低碳产品认证试点，建立节能环保名优产品目录。设立碳交易平台，办好成都碳交易市场。

（八）着力深化重点领域改革，加快建设最具活力城市

一是深化供给侧结构性改革。淘汰落后和过剩产能，防止新增过剩产能，扩大有效产能。加强精准调控，构建房地产市场平稳发展长效机制。加强面向民营企业、中小微企业的金融创新，强化金融风险防控。促进电力市场化交易，推动大用户直购电，降低企业制度性交易成本及税负、人力和物流等成本，切实减轻企业负担。

二是深化统筹城乡改革。深化集体经营性建设用地、农村金融、农民财产性收入改革试点。完善来蓉人员落户制度，推行居住证积分入户与条件入户双轨并行。继续推进和完善不动产统一登记。加快幸福美丽新村建设，集中连片规划建设“小组微生”新农村综合体，新改建村组道路1600公里。深化村级公共服务和社会管理改革，加快建设一批统筹城乡综合改革示范镇。

三是深化国资国企改革。加快推动市属国企改革整合转型，积极推进混合所有制改革，实行分类监管考核，出台市属国企强化审计监督、推进经营管理层市场化选聘、加强专职外部董事队伍建设等制度，开展市属国企“压层级、减数量、控新增”专项行动，实现2户以上市属国企上市。

四是深化财税和投融资体制改革。调整优化转移支付结构，增加一般性转移支付规模和比例，严格实施预算中期评估和绩效评价管理，完善政府债务管控机制。建立统一规范的税收第三方信息交换平台和信息共享机制，强化纳税信用等级评定，加大税收违法联合惩戒力度。全面落实鼓励民间投资各项政策，缩减核准事项，完善投资项目“三个清单”管理制度，拓宽融资渠道，充分激发社会投资活力。

（九）着力持续保障改善民生，加快建设健康幸福城市

一是实施健康成都战略。坚持健康优先，加快推进国家健康城市试点建设，强化“三医”联动，加强医联体内涵建设和家庭医生签约服务，完善分级诊疗制度。实施“互联网+医疗”行动，推动医疗机构间信息共享、检验检查结果互认，探索建立市民全生命周期健康档案系统。加快健康养老产业发展，统筹推进成都医学城等园区建设。大力发展中医药产业，提升中医药健康服务能力。全面贯彻人口计生法。推进医养结合，加快社区养老院建设，加大空巢老人关爱力度，推动建立多层次普惠制养老服务体系。积极开展全民健身，实施市民体质提升计划，加快推进区（市）县“一场一馆一池”“两中心”建设。

二是继续抓好脱贫攻坚。加快推进简阳市脱贫攻坚，大力开展对口帮扶，全面落实各项扶贫政策，新建贫困村农业园区32个，完成易地搬迁贫困群众10794人，确保58个省定贫困村全部摘帽、31112名贫困人口全部脱贫。扎实抓好甘孜、阿坝19个县对口支援，推动新一轮对口援藏五年规划，深化文旅、农业务实合作，共同推动成甘、成阿工业园区加快建设。高标准推进城乡精准扶贫，完成第三轮第二批“百村万户”帮扶行动，深化城市下岗职工、支出型贫困、低保边缘群众等群体扶贫，让贫困群众感受到党和政府的温暖，感受到全社会的关心。

三是抓好就业和社会保障。实施更加积极的就业创业政策，加强职业能力和“技能成都”建设，创建中国（成都）职业训练院、中国（成都）创业公共实训基地，加快完善职业培训体系，推进重点群体就业创业。完善城乡社会保险制度体系，建立健全参保缴费激励约束机制和待遇调整机制，全面实施重特大疾病医疗保险制度，稳步推进“两定”机构协议管理。加快构建租售补并举的住房保障体系，继续推进住房公积金制度扩面。健全社会救助体系，建立社会关爱援助体系，强化农村留守儿童关爱保护，大力实施助残“三大工程”。

四是推进教育品质不断提升。深化教育综合改革，进一步推进义务教育优质均衡发展，建成公办幼儿园160所，新增学位4.8万个，新建和改扩建中小学60所，新增学位3.2万个。实施普通高中优质特色发展计划，加快发展现代职业教育体系，规划筹建成都艺术学院，高水平办好成都大学。扎实抓好民办教育分类管理，积极推进社区教育和老年教育，加大特殊教育支持力度。大力发展智慧教育，打造教育优质资源云平台，让每一个孩子都有机会享受到优质的教育。

五是深化平安成都建设。健全落实安全生产责任体系，加强风险管控和隐患排查治理，强化道路交通、建筑施工、危化品、特种设备等重点行业领域安全监管，切实做好地质灾害防治和防汛工作，强化人员密集和易燃易爆场所消防安全。加快建设立体化、信息化社会治安防控体系，充分接入和利用社会面资源，提升社会治安监控覆盖率，健全突发安全事件联动平台，增强公共安全预警预防和综合应对能力。深入推进信访制度改革，按照“三到位、一处理”的要求，及时解决群众合法合理诉求。积极支持国防和军队改革，加强国防动员和后备力量建设。

我们将坚持有感发展，以群众最急最愿最盼为先，办好交通出行、市容环境、就业创业、扶贫济困、教育医疗、养老服务、食品安全、百姓安居、文体惠民、便民服务十大民生实事，让城市更美好，市民更舒心。

三、关于政府自身建设

加快建设国家中心城市，迫切需要我们主动顺应时代发展新趋势，准确把握经济社会转型新要求，不断满足人民群众新期待，提升特大城市治理体系和治理能力现代化水平，加快建设有限、有效、有为的现代政府。

（一）全面建设学习型政府。强化理论武装，坚持用中国特色社会主义理论体系武装头脑，深入学习习近平总书记系列重要讲话精神和治国理政新理念新思想新战略。强化能力提升，努力学习现代市场经济、信息技术、城市治理和国际规则等方面知识，增强战略创新思维能力，着力提升专业化水平。强化智库建设，搭建聚智平台，加强调查研究，跟踪研究时代前沿科技动态，借鉴世界先进城市治理经验，强化政府核心智库战略谋划、咨政建言、政策评估和汇智聚智等功能，更好服务政府科学决策。

（二）深入推进依法行政。严格执行市人大及其常委会决议决定，自觉接受人大及其常委会工作监督、法律监督和政协民主监督。切实强化依法决策，出台法治政府建设实施方案，抓好重点领域行政立法；健全完善重大行政决策法定程序、“三重一大”事项决策机制和政府法律顾问制度。全面推进综合执法，推进跨部门跨领域综合行政执法改革试点，实施执法事项属地化管理；健全重大执法决定法制审核、执法全过程记录、行政执法公示等制度。强化基层依法治理，推广“大联动·微治理”经验，抓好“法律七进”，深化城乡社区可持续总体营造行动。

（三）加快转变政府职能。深入推进简政放权，推行市场准入负面清单制度，全面清理各类不合法不合理证明，在区（市）县全面推行相对集中行政许可权改革，实现“一颗印章管审批”。积极推行工程建设领域“多评合一”“联合验收”等新模式。全面深化商事制度改革。加强和创新重点领域市场监管，推行“双随机、一公开”监管，健全失信惩戒制度，投资项目审批管理平台实现全流程监管和挂牌督办，切实提高政府监管效能。着力建设“品质成都”，实施品质提升行动，开展市长质量奖评选，深化国家农产品质量安全市建设，创建国家食品安全示范城市。加快政府管理服务标准化建设，实现行政审批标准化管理、公共资源交易标准化作业，推动各行业各领域采用国际一流标准。深化政府购买服务改革，年度政府采购预算总额35%以上份额，面向中小微企业特别是创新创业企业采购；推广科技企业创新券等凭单制购买方式。

（四）全面推进网络理政。坚持上网访民意、下网解民忧，加强与群众、企业沟通交流，寻求社会“最大公约数”。推进B2G政企、R2G政民新型互动平台和S2G公共安全政府服务平台“三网融合”，着力打造全媒体多功能一体化网络理政系统。全面推进政务公开，做好政策解读，推进公开内容覆盖权力运行全流程，实现政务服务和行政审批中介服务事项全面公开；健全市长公开电话、网络信箱、短信、微信、移动客户端服务体系，实现12345平台与市级公共服务单位热线电话“一键通”；及时回应政务舆情，提高政务“两微一端”开通率和回应信息到达率。全面推进网上服务平台和办公平台一体化建设，建成4G无线政务专网和多平台终端移动办公体系，基本建成数据共享交换平台和政务服务信息系统，率先实现工商登记、行政审批、行政执法等重点信息共享共用，加快推进公共数据资源向社会开放，电子证照流转交换与网上身份认证体系投入应用，部门审批事项全部接入网上政务大厅，基本公共服务事项90%以上可在网上办理，初步实现政务服务和协同办公的跨区域、跨层级、跨部门“一号申批、一窗处理、一网通办”，让政府服务更贴心、市民办事更省心。

（五）切实强化廉洁政府建设。加强重点领域和关键环节审计，规范财政资金分配使用管理。坚持“权力就是责任、责任就要担当”的理念，以更大力度、更严要求正风肃纪。健全政策落实的责任机制、奖勤罚懒的激励机制、常态长效的问责机制，强化“大督查”“督考合一”，优化考评结果运用。大力倡导崇尚事业、追求卓越的价值取向，弘扬兢兢业业、精益求精的工匠精神，秉持雷厉风行、真抓实干的作风品牌，健全容错纠错免责机制，为担当者担当、为负责者负责、为改革创新者撑腰鼓劲，让广大干部愉快工作、健康生活。

各位代表！梦想照亮前方，奋进正当其时。成都发展已站在新的历史起点上，让我们紧密团结在以习近平同志为核心的党中央周围，在党中央国务院、省委省政府和市委的坚强领导下，团结带领全市人民，拼搏实干、科学发展，为持续增进民生福祉、加快建设国家中心城市而努力奋斗！
